# Round the Hospitals

**Published:** 1918-12-07

**Authors:** 


### ROUND THE HOSPITALS.

DURING their visit to Edinburgh the King and Queen had an interesting encounter with some of the sailor and soldier patients from Kingscroft Hospital, Barnton, who were at the station with their matron, Miss Hilda M. Smith. Their Majesties, who were accompanied by the Prince of Wales, were greatly interested in the little group, and found time to linger with them and hear some individual

**ROUND THE HOSPITALS**—*(continued)*.

stories of the war. Miss Hilda Smith was presented to Their Majesties, and the Queen remarked upon her 1914 Star and learned that she had been nursing British soldiers in France for three years and a half before taking up her present post. The Queen expressed her regret that she could not visit the hospital, which is the first opened in Scotland for discharged sailors and soldiers needing medical attention.

PRINCESS MARY'S tour in France and Flanders, completed just as the King and her brothers were arriving, was a brilliant success from start to finish. She carried out her original plan in its entirety, and besides visiting Abbeville, Rouen, Trouville, Dieppe, Le Tréport, Dunkirk, and St. Omer, she fitted in an extra expedition to Bruges and Ypres. At the latter place she witnessed in the market-place the march past of her own regiment, the Royal Scots. In the course of her travels the Princess went over many hospitals and rest stations, and saw various forms of war organisations, such as ordnance camps, camouflage parks, and signalling depôts. She had a very pretty welcome home at Victoria, where she arrived at about 7 P.M. on November 29. A guard of honour, consisting of members of her own and other detachments was drawn up on the platform to receive her, together with members of the Q.M.A.A.C., and the Princess, who was in her V.A.D. uniform, inspected the group on the invitation of the Commandant, and shook hands with the officers of the Corps. To add to the enthusiasm the soldiers from the train, returning on leave, gathered to cheer her as she drove off, and the Princess was visibly delighted with her reception.

COMMANDANTS and matrons of Red Cross hospitals are becoming a little anxious as the time draws near for returning loans to their owners. There are not many articles, even of metal, which do not show signs of wear after some years even of fair use in a military hospital, and between the too cheerful patient bent on creating a sensation and the man with a grievance who will go the length of burning the inside of a piano it has gone rather hard with the furniture in some establishments. At Gloucester the Commandant is writing to warn the owners of loaned articles that "*if existent at all*" they will certainly show signs of wear and tear.

WE understand that no instructions have yet been received from the military authorities as to the fate of the auxiliary hospitals. It is expected that they will be closed down gradually. In some localities they will be badly missed by their voluntary staff, who have given the best part of their time for years to this work. A movement in favour of founding a cottage hospital as a war memorial in such places as have no hospital within easy reach has been started, but such enterprises are costly, and they demand a highly specialised type of administrative ability usually lacking in small country towns. What is wanted in the country is a series of clinics, where sick children and babies could have in-patient treatment and care with accommodation for maternity patients. We should like to see a great extension of this necessary work, which can be attached to a welfare centre without stirring any of the intricate problems involved in the cottage hospital.

THE QUEEN has given a beautiful Communion Service to the hospital chapel of the Queen's Hospital at Sidcup, welcomed as yet another mark of "the sympathy and interest always displayed by Her Majesty in the institution," and as "evidence of a particularly kind and gracious thought." It is delightful to trace the constant marks of what may be called special pride in ownership displayed by the Queen towards the institutions which bear her name. Too often such links are regarded as purely formal, and represent no individual interest. But the Queen recognises the mystic symbolism of a name, and uses it as occasion for service.

AT the monthly meeting of the Northampton County Red Cross and St. John Ambulance Brigade Committee it was reported that the total number of patients received by convoy in the county hospitals totalled over 20,000 to date. The future of the organisation was discussed, and a proposal for treating sailors and soldiers suffering from tuberculosis, of whom it is estimated there are at least 25,000, was reported. Each county is being asked to retain two hospitals to deal with these patients, one for men in the advanced stages and the other, in the nature of a farm colony, for early and light cases. In the latter class of institution there will be plenty of room for Voluntary Aid workers, especially such as have some experience on the land.

THE Hospital Supplies Depôt, opened in the Guildhall, Hull, over three years ago, is still running, and the demands upon it have not yet begun to slacken. It has sent out nearly 100,000 articles during its short existence, the work of 254 ladies enrolled for this purpose. The list of hospital requisites prepared by these zealous workers comprises exactly such things as are needed by all institutions for the sick. It would be a public calamity if the services of the Hull Depôt were lost to the institutions in its own county, and we cannot but hope that Mrs. Oswald Sanderson, who has been chairman since the work started, will examine into the needs of the voluntary hospitals in the county before closing down.

ALTHOUGH in many directions the American Red Cross is still working at high pressure, the manufacture of all surgical dressings at the work-rooms in Grosvenor Gardens is to cease this week. There are now sufficient surgical dressings and appliances for all possible requirements, and their preparation has been discontinued throughout America. Nurses, however, are being still enrolled in the United States for post-war work in the various countries

**ROUND THE HOSPITALS—(*continued*).**

which need their help. Among the places where the Red Cross nurses are helping to bring about a real social regeneration is Jerusalem.

At the York County Hospital some satisfactory alterations are being made in the salaries of the higher grade members of the nursing staff. The assistant matron is to have £70 a year, the night sister £60, the ward sisters £50, rising by yearly increments of £5 to £60, and receiving thereafter such increase as may be recommended by the matron to the committee.

Much of vital interest to matrons and sisters in all branches of nursing lies buried in reports, inaccessible to the actual workers. This is specially true of the volume in which Sir George Newman, Chief Medical Officer of the Board of Education, reports on the past year's work and advises on the future. He wants nurses and the public to avoid concentrating on the child with defects and diseases, and to turn their thoughts to improving the standard of health in the whole child population. His figures are very remarkable. He finds that 10 per cent. of the children in the country and only 7 per cent. in London are absent from school owing to more or less chronic ill-health. In London 40 and in the country 65 per cent. of the children have carious teeth; in London 5 and in the country 21 per cent. have disease of nose and throat; in London 2 and in the country 8 per cent. have verminous heads. The figures point to the success of the preventive work so splendidly organised in London, so intermittent in the country.

Contrary to what everyone has been led to believe, the children have not suffered from malnutrition during the war, nor have their nerves been shattered during the raid season. The children, in fact, were considerably better nourished, so far as averages can show, in 1917 than in 1913. There is no evidence at all that any general strain has fallen on the mass of children owing to the air-raids, though some 600 boys and over 700 girls were reported as "suffering from nervous complaints." This refreshing news reflects high credit on the rationing authorities and on the nerve stability of the English race.

The opening of the new Nurses' Home at the West London Hospital puts the crown on many years of strenuous effort. We offer sincere congratulations to the matron, Miss Nevile, that it has been found possible to complete the building and equipment of Abercorn House during the war. The fact speaks to the urgency of the need. Shortly before war broke out we had the painful duty of inspecting the accommodation offered to nurses at the West London Hospital, and we then reached the conclusion that nothing but an uncommon spirit of mutual trust and friendliness, extending from the highest to the lowest, under a conspicuously able matron, could have kept the staff working harmoniously together in the teeth of the real privations they were called on to endure. We trust many of the sisters and nurses who carried on so cheerfully under the old conditions are still connected with the West London and are enjoying the delightful contrast. We are glad to hear there is a nurses' dispensary as well as a good sick bay. It is very satisfactory also to learn that the roof has been constructed with a view to adding another storey if (we would rather say when) more accommodation is required.

An interesting report on nurses' hours has been issued in Melbourne by the Chief Clerk of the Treasury, who was charged with the task of investigating various charges of over-working nurses which have been levelled against the public hospitals. It appeared that the hours on duty, exclusive of meals, varies in the public hospitals respectively from ten and a half to eight by day, and from eleven to seven and three-quarters by night. It was recommended that the eight-hour system should be tried, and that a record of the hours actually worked by the nurses should always be kept and be produced if required by the Inspector of Charities. It was also recommended that a conference of matrons, superintendents, and metropolitan hospitals should be held, with a view to preparing a uniform curriculum, and that nurses be relieved as far as possible from the performance of purely domestic duties.

But long before any conference could be held an indignant chorus of remonstrance has issued from the Melbourne hospitals affected by the above recommendations. The Melbourne Hospital authorities estimate that it would take fifty-six extra nurses and ten extra domestics to carry them out, involving a capital expenditure of £20,000 to £30,000, and an annual cost of £5,000. The Nurses' Committee maintains, however, that this estimate is grossly exaggerated, and that the alteration could be effected with thirty-two extra nurses. This committee comprises many of the nurses at the Melbourne Hospital and other institutions in the town, and it aims, among other things, at abolishing the fourth year of training. Nurses have certainly found a voice on the other side of the world, but that is not quite the same thing as using it to the best advantage.

Influenza has been raging in India beyond anything hitherto reported of European countries. In Bombay city alone there have been 15,000 deaths, and in every town and country district throughout the Punjab from 5 to 10 per cent. of the population have fallen victims to the disease. To say that the Nursing Service has broken down would be ridiculously to understate the case. No Nursing Service exists in the country districts, and patients have no means of obtaining medical aid. It is estimated by the *Times* that if the death-rate from influenza throughout India should equal that in the Punjab, it means a total death-roll of over three million people.

**ROUND THE HOSPITALS**—(*continued*).

A remarkable account of Miss Edith Cavell is published in the *Daily Telegraph* of November 27. It is the story of the Rev. H. S. T. Gahan, the British chaplain, who ministered with fine devotion all through the long years of war to the little British community of some 900 persons in Brussels, mostly women and elderly men. We hope to give the narrative as it stands, and we think few will be able to read it without tears. We know no record more vividly presenting a life hid with Christ in God. The selflessness which even in the moment of supreme ordeal could reject the comfort of a compatriot's presence because "it would be too much for him" was a spontaneous and thoroughly characteristic impulse of one accustomed to consider always in what way pain might react upon her friends. And the meeting when it took place shows us the pure gold of Edith Cavell's nature exposed without the smallest tincture of any lower level of consciousness. "I have never seen anyone more perfectly sweet, composed and quiet. It was just as if she were about to go to bed to get up the next morning and go about her ordinary life."

---

The Ranyard nurses have had the high satisfaction of seeing the war end before the celebration of their jubilee. Fifty years ago three or four Ranyard nurses began visiting the sick poor of London. Now their number has swelled to eighty-three, and still the demand for workers outgrows the supply. The Archdeacon of Westminster, who spoke at the concert at the Æolian Hall given on November 22 on behalf of the Ranyard Nurse Fund, made a special appeal for women now relinquishing war work to dedicate their services to the poor of London. "The work calls for a true spirit of adventure and a grip of social problems, as well as a heart of sympathy and understanding." Princess Louise, Duchess of Argyll, has sent £50 to the fund with the assurance of her heart's sympathy, and £150 was realised at the concert. Ranyard nurses have always upheld a high standard of training, and the recruits they want are women of vision.

---

It is much to be desired that the sailors and soldiers who have passed through the hospitals should become articulate and let the public understand what is in their mind. We have been much struck with the suggestions of Lance-Corporal Rhodes, of the Gledhow Hospital, Leeds, on the subject of a memorial to the Voluntary Aid Detachments. He wants to see pleasant homes for the permanently disabled established within easy reach of every large town, where the men could be comfortably housed, learn such trades as were suited to their infirmities, and still remain under the care of voluntary workers. He would like the letters V.A.D. inscribed on the buildings, even though they be maintained by the State, and there is reason to hope that some really good scheme of co-operation between voluntary workers and the State may be evolved.

Welfare workers are just beginning to understand the soothing and curative effects of land work on neurasthenics. Many girl munition workers whose health has suffered under the strain have been transferred to farm work under suitable conditions, and have made a brilliant recovery. But for this chance of recovering their health it is believed many would have been invalids for life. A centre started for the treatment on land work lines of hospital nurses exhausted by over-long hours of confinement would, we believe, prove a real boon to many. The regular occupation, open-air life, freedom from worry, and possibly some occult healing power in contact with Nature, seem able to restore the tired nerves, which drugs and confinement to bed with intensive feeding often appear merely to aggravate.

---

Queen Victoria's Jubilee Institute for Nurses, Rutland Square, Dublin, is justly proud of the record of work accomplished by the nurses during the run of the influenza epidemic. Many of the sick poor of Dublin are blessing them for their sympathy and help. In the month of October six nurses visited 317 cases, and paid 3,414 visits. Of the cases 269 were influenza and pneumonia. Two of the nurses contracted the epidemic, leaving the burden still heavier on the remainder of the staff. The only outside help was from two V.A.D.s, who generously came to their assistance. It is urged locally that St. Lawrence's Home should have a staff of twelve nurses at least, but want of funds has obliged the committee to reduce the number to six. If only the public would give more help to the committee the work could be extended, to the great advantage of the poor of the city.

---

The unhappy woman who dressed herself up for fraudulent purposes as a Red Cross nurse, and positively provided herself with a sham wounded arm and a sham limp, was probably not capable of appreciating the baseness of her conduct. It is satisfactory to learn that she was promptly detected, and is now removed from the temptation of repeating the offence for some months to come. But there should be close inquiry into the manner in which this old offender obtained possession of the uniform and badges, together with the M.M. and D.C.M. ribands. A great many Red Cross nurses will shortly be discarding their uniform, and it is a matter of some concern to the public that these second-hand garments and badges should not be offered for sale.

---

☛ It is our invariable practice to pay for all items of news which we may publish. The minimum payment is 5s. Paragraphs of about twenty lines in length—that is to say, of 150 words or less—are preferred. Contributions should be addressed to The Editor, The Hospital, 28 and 29 Southampton Street, Strand, London, W.C. 2, and, to be in time for the current week's issue, should reach him by the first post on Mondays.

